# *In situ* cellular immune response in non-ulcerated skin lesions due to *Leishmania (L.) infantum chagasi* infection

**DOI:** 10.1590/1678-9199-JVATITD-2020-0149

**Published:** 2021-02-26

**Authors:** Carmen Sandoval, Gabriela Araujo, Wilfredo Sosa, Sara Avalos, Fernando Silveira, Carlos Corbett, Concepción Zúniga, Marcia Laurenti

**Affiliations:** 1 Laboratory of Infectious Diseases Pathology, Department of Pathology, Medical School (FMUSP), University of São Paulo (USP), São Paulo, SP, Brazil.; 2 Microbiology Research Institute, National Autonomous University of Honduras, Tegucigalpa, Honduras.; 3 Master Program in Infectious and Zoonotic diseases, School of Microbiology, National Autonomous University of Honduras, Tegucigalpa, Honduras.; 4 Department of Parasitology, Evandro Chagas Institute, Secretariat of Health Surveillance, Ministry of Health, Belém, PA, Brazil.; 5 Institute of Tropical Medicine, Federal University of Pará, Belém, PA, Brazil.; 6 Department of Health Surveillance, School Hospital, Tegucigalpa, Honduras.

**Keywords:** Non-ulcerated cutaneous leishmaniasis, Atypical cutaneous leishmaniasis, Leishmania infantumchagasi, Cellular immune response, Immunohistochemistry, Honduras

## Abstract

**Background:**

Skin lesions of patients affected by non-ulcerated cutaneous leishmaniasis (NUCL) caused by *L. (L.) infantum chagasi* are characterized by lymphohistiocytic inflammatory infiltrate associated with epithelioid granuloma and scarce parasitism. However, the *in situ* cellular immune response of these patients is unclear. Therefore, the aim of the present study was to characterize the cellular immune response in the skin lesions of patients affected by NUCL.

**Methods:**

Twenty biopsies were processed by immunohistochemistry using primary antibodies to T lymphocytes (CD4, CD8), NK cells, B lymphocytes, macrophages, nitric oxide synthase and interferon-gamma.

**Results:**

Immunohistochemistry revealed higher expression of all cellular types and molecules (IFN-γ, iNOS) in the dermis of diseased skin compared to the skin of healthy individuals (p < 0.05). Morphometric analysis performed in the skin lesions sections showed the predominance of CD8^+^ T lymphocytes in the mononuclear infiltrate, followed by macrophages, mostly iNOS^+^, a response that could be mediated by IFN-γ.

**Conclusion:**

Our study improves knowledge of the cellular immune response in non-ulcerated or atypical cutaneous leishmaniasis caused by *L. (L.) infantum chagasi* in Central America and pointed to the pivotal participation of CD8^+^ T lymphocytes in the host defense mechanisms against the parasite in patients with NUCL.

## Background

*Leishmania* (*L*.) *infantum chagasi* is etiological agent of visceral leishmaniasis (VL) in America. However, in some countries of Central America - including Costa Rica, Nicaragua, El Salvador and Honduras - this parasite specie besides VL also causes atypical or non-ulcerated cutaneous leishmaniasis (NUCL) [1-5]. It is important to mention that both VL and NUCL do not occur at the same time in the same patient. Usually NUCL affects children over 5 years old and young adults and VL children under 5 years old [4,6].

NUCL have been reported since the 1990s in Honduras, when an increased number of non-ulcerated lesions was observed in endemic areas of *L. (L.) infantum chagasi* transmission. Non-ulcerated nodules or papules of small size, generally surrounded by a hypopigmented halo, mainly located in exposed areas of the body, and habitually of chronic evolution, characterize these cutaneous lesions. Campos-Ponce et al. [7] suggest that the *L.* (*L*.) *infantum chagasi* tropism to viscera or skin observed in Honduras could be strongly related to the immunological background of the patients, since genotypic differences among parasites strains were reported [1,2]. It must be highlighted that cutaneous lesions caused by *L. (L.) infantum chagasi* have already been described in South America, but the reports related to ulcerated lesions are different from those observed in Central America, which independently of the time of evolution do not ulcerate [8-11].

Microscopically, NUCL skin lesions are characterized by a mononuclear inflammatory infiltrate in the dermis of variable intensity, formed mainly by lymphocytes and macrophages, associated with epithelioid granuloma and scarce parasitism. The epidermis remains preserved and only slight thinning is observed in some cases [12]. Among to lymphocytes subpopulation, the involvement of the Th17 cells in NUCL skin lesion has recently been described [13], indicating that the presence of Th17 lymphocytes could play a pro-inflammatory role promoting the control of tissue parasitism. However, despite the participation of the Th17 inflammatory response, the total control of tissue parasitism and the spontaneous healing of the lesion does not occur; probably, due the participation of regulatory immune response (FoxP3^+^ cells), mainly through the production of TGF-β [14].

There are scarce reports relating histopathological features or cellular immune response of the skin lesion caused by *L.* (*L*.) *infantum chagasi* in America. Therefore, to deepen our knowledge on aspects of *in situ* cellular immunity to better understand the clinical aspects of disease, the present study characterized CD4 and CD8 T lymphocytes, B lymphocytes, NK cells, macrophages, as well as elements of activation such as IFN-γ and iNOS in skin lesions of patients with non-ulcerated cutaneous leishmaniasis provoked by *L. (L.) infantum chagasi* using immunohistochemistry.

## Methods

### Patients and samples

**Figure 1.**
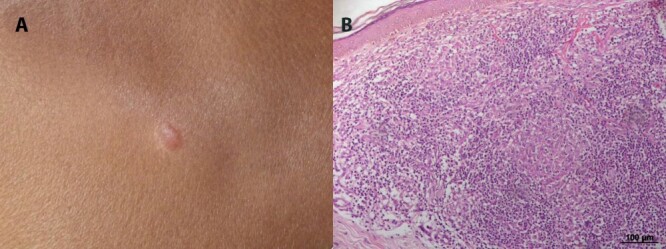
Clinical feature of the skin lesion of non-ulcerated or atypical cutaneous leishmaniasis. **(A)** Macroscopic lesion. **(B)** Histological sections showing the mononuclear inflammatory infiltrate in the dermis.

Twenty biopsies of skin lesions from patients with NUCL from an area of *L. (L.) infantum chagasi* transmission located in the south Honduras, municipalities of Amapala and Orocuina, were used. None of the patients included in this study had a previous history of VL, nor received treatment for leishmaniasis. All patients presented positive parasitological diagnosis in scrapings of lesions stained by Giemsa, and *L.* (*L*.) *infantum chagasi* was characterized by RT-PCR [12]. The present study was approved by two institutional ethics committees (research protocols #03-2014 and #051/15). Informed consent was obtained from all participants in the study. As already reported [12], from 20 NUCL patients, 100% presented non-ulcerated cutaneous lesions (Figure 1), 65% were women and 20% were men. Gender was not reported in 15% of the patients. The average age was 33.4 years, ranging from 9 to 70 years. The evolution time of lesions varied from 1 to 240 months. About 70% of the lesions had a diameter of 3 to 5 mm, and the majority were unique. The lesions were located on the extremities (arms and legs) or face. After diagnosis and biopsies collection, all patients included in this study received treatment according to the protocol handled by the Ministry of Health of Honduras [3].

### Immunohistochemistry study

The *in situ* cellular immune response was characterized by immunohistochemistry. Briefly, we performed the tissue deparaffinization and hydration, blockade of the endogenous peroxidase in 3% hydrogen peroxide, and antigen recovery using citrate buffer (10 mM/pH 6.0) in a boiling water bath for 30 min. After, the samples were incubated overnight at 4 °C with the following primary antibodies: CD4 (monoclonal, NCL-L-CD4-1F6, Novocastra), CD8 (monoclonal, NCL-L-CD8-295, Novocastra), CD20 (polyclonal, (C-20): SC-7733, Santa Cruz Biotechnology), CD56 (monoclonal, NCL-L-CD56-504, Novocastra), CD68 (monoclonal, ab955, ABCAM), iNOS (polyclonal, (N-20): SC-651, Santa Cruz Biotechnology) and IFN-γ (polyclonal, (H-145): SC-8308, Santa Cruz Biotechnology) at 1:20, 1:100, 1:1000, 1:100, 1:400, 1:200 and 1:100 dilutions, respectively. Isotype controls were used as negative controls. For all markers, the Novolink Kit (RE7280-K-Leica) was used. All reactions were developed using a chromogenic substrate, DAB+H_2_O_2_ (diaminobenzidine with hydrogen peroxide-K3468-Dako Cytomation), followed by Harris haematoxylin counterstaining. Finally, the slides were dehydrated in a series of ascending alcohols and mounted with Permount and a glass coverslip. Tonsil sections were used as positive controls. Cells marked in brown were considered positive.

Ten skin samples obtained from healthy individuals undergoing plastic surgery were included as controls.

### Quantitative morphometric analysis of immunostained cells

Ten sequential fields of each histological section (objective 40×) were photographed in an optical microscope coupled to the computer using the AxioVision 4.8 software (Zeiss). Brown-immunostained cells were quantified, taking into account the cell colour pattern and morphology for each antibody using ImageJ software. The determination of the cell density (cells per square millimeter) of each marker was calculated by the ratio between the immunostained cells and the area of each photo.

### Statistical analysis

Statistical analysis was performed with GraphPad Prism 5.0 software. We used the Kolmogorov-Smirnov test to normality test, the t-test for data with a Gaussian distribution, the Mann-Whitney test for those without normal distribution, and the Spearman and Pearson tests to correlate the different immunohistochemical markers.

## Results

### Immunohistochemical analysis

**Figure 2.**
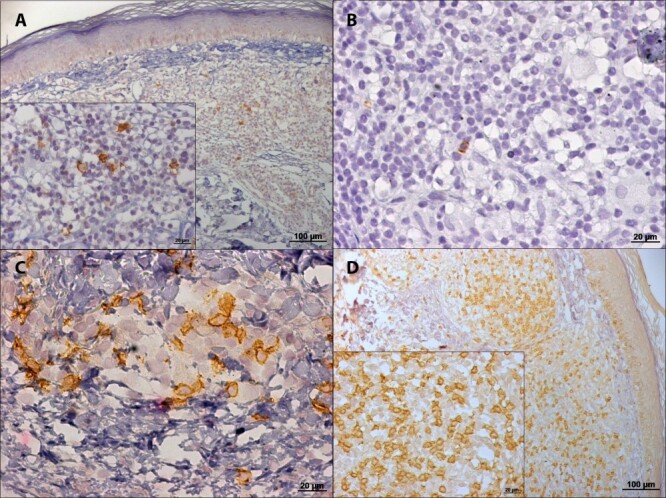
Photomicrographs of immunohistochemistry reaction in histological sections of skin lesions of patients with non-ulcerated or atypical cutaneous leishmaniasis showing in the dermal inflammatory infiltrate: **(A)** B lymphocytes (CD20^+^); **(B)** NK cells (CD56^+^); **(C)** CD4^+^ T lymphocytes and **(D)** CD8^+^ T lymphocytes.

**Figure 3.**
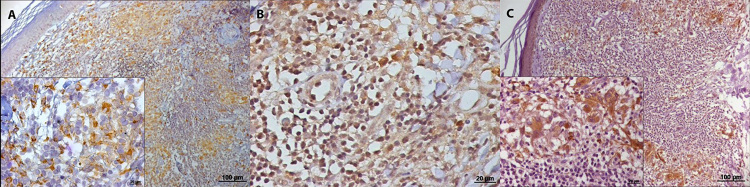
Photomicrographs of immunohistochemitry reaction in histological sections of skin lesions of patients with non-ulcerated or atypical cutaneous leishmaniasis showing in the dermal inflammatory infiltrate: **(A)** macrophages (CD68^+^); **(B)** IFN-γ^+^ and **(C)** iNOS^+^ cells.

The skin lesions of patients with NUCL showed the presence of all the markers in the dermal mononuclear infiltrate, B lymphocytes (CD20^+^), CD4^+^ and CD8^+^ T lymphocytes, NK cells (CD56^+^), macrophages (CD68^+^), iNOS^+^ and IFN-γ^+^ cells (Figures 2 and 3).

**Figure 4.**
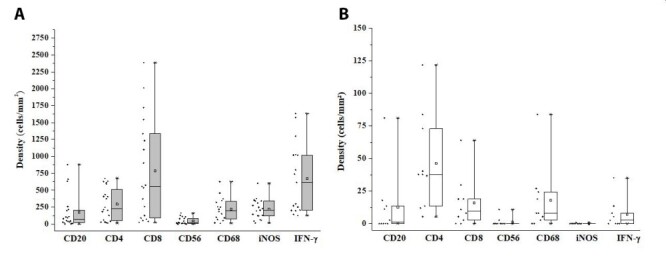
Dot plot graph showing the distribution of positive cells and a box plot showing the median, mean, quartiles, maximum and minimum values for the number of positive cells per square millimetre for CD20, CD4, CD8, CD56, CD68, iNOS and IFN-γ markers in **(A)** skin lesion of non-ulcerated cutaneous leishmaniasis and **(B)** skin of healthy individuals.

The quantitative analysis showed that the cellular density (mean ± standard error) was 173.2 ± 56.4 for CD20^+^ cells, 785.8 ± 169.7 for CD8^+^ T lymphocytes, 296.6 ± 53.5 for CD4^+^ T lymphocytes, 47.8 ± 12.7 for CD56^+^ cells, 219.9 ± 43.2 for CD68^+^ cells, 219.5 ± 33.7 for iNOS^+^ cells and 671.4 ± 124.9 cells/mm^2^ for IFN-γ^+^ cells in the skin lesion of patients affected by NUCL (Figure 4A). In contrast, the cellular density of all markers in the skin of healthy individuals was lower than their observed in the skin lesions of individuals with NUCL (p < 0.05). So, the cellular density in health skin was 12.7 ± 7.9 for CD20^+^ cells, 15.9 ± 6.1 for CD8^+^ T lymphocytes, 46.2 ± 11.6 for CD4^+^ T lymphocytes, 1.4 ± 1.1 for CD56^+^ cells, 17.9 ± 8 for CD68^+^ cells, 0.1± 0.1 for iNOS^+^ cells and 7.2 ± 3.8 cells/mm^2^ for IFN-γ^+^ cells (Figure 4B).

For better understanding the participation of the cellular immune response in the inflammatory infiltrate, the ratio between CD4 and CD8 T lymphocytes, as well as iNOS^+^ cells and macrophages (CD68) in the lesions of patients and healthy skin was evaluated. The ratio of CD4/CD8 was 8 times lower in the cutaneous lesions of NUCL patients (0.38) compared to healthy skin (2.92), pointing to the high participation of T CD8^+^ lymphocytes in the NUCL lesions. On the other hand, the ratio of iNOS/CD68 was 167 times higher in NUCL (1.0) than that observed in healthy skin (0.006). Considering NUCL, the ratio of IFN-γ/CD8 was 0.85, IFN-γ/CD4 was 2.26, and IFN-γ/CD56 was 14.04, suggesting that the majority of IFN-γ-producing cells are CD8 lymphocytes.

A positive correlation was observed among all the cellular markers used in this study, except for NK cells (CD56). Taking the most important correlations, a positive and strong correlation was observed between the density of CD8^+^ T lymphocytes and IFN-γ^+^ cells (*r*_*s*_ = 0.8794 and p = 0.00001); and also between CD4^+^ T lymphocytes and IFN-γ^+^ cells (*r*_*s*_ = 0.7206 and p = 0.0011). The density of macrophages (CD68^+^) showed a positive and strong correlation with the density of iNOS^+^ cells (*ρ* = 0.8909 and p = 0.00001), and a positive and moderate correlation was also observed between iNOS^+^ cells and IFN-γ^+^ cells (*r*_*s*_ = 0.6520 and p = 0.00621).

## Discussion

The *in situ* cellular immune response in cutaneous leishmaniasis caused by dermotropic strains has been widely studied. Immunological spectrum of cutaneous leishmaniasis has been observed in human disease varying from a strong T-cell response characterized by hypersensitivity with high TNF-α and IFN-γ production, with subsequent activation of macrophages and lysis of the parasite; to the lack of delayed hypersensitivity response (DTH) characterized by a Th2-type immune response, with high production of IL-4, IL-10 and TGF-β, leading to suppression of T cells and disease progression [15-17].

*L.* (*L*.) *infantum chagasi* is classically causative agent of visceral leishmaniasis, but in some regions skin lesions caused by this specie of parasite have been reported in patients with no previous history of VL. However, the clinical presentation varies depending on the region. Reports has been shown that in the Old World, the most usual clinical feature is a single lesion that consists of small, crusty ulcers surrounded by a notable erythematous reaction or non-ulcerated papules that, when ulcerated, are recovered with a discrete crust [18-22]. In some countries of South America, these lesions clinically can be papular, nodular, or ulcerated with or without the presence of crust [8-11]. However, in Central America [4,12-14,23], the patients presented small nodular lesions that do not cause ulcer independent of the evolution time of infection.

The histopathological features reported in Europe by *L*. (*L*.) *infantum* infection are characterized by slight hyperkeratosis with parakeratosis and moderate acanthosis in the epidermis; and in the dermis, the findings depend on the time of evolution of infection, in the initial phase the inflammatory infiltrate is dense and diffuse composed of parasitized histiocytes, lymphocytes, and plasma cells with a small number of eosinophils and neutrophils, in chronic lesions the inflammatory infiltrate is granulomatous, with epithelioid cells and multinucleated giant cells, with a reduction in the number of parasites [18-20,24]. On the other hand, the histopathological changes of epidermis in non-ulcerated skin lesion caused by *L. (L.) infantum chagasi* in Central America is characterized by slight thinning, mild acanthosis, and focal lymphohistiocytic exocytosis in 40%, 10% and 20% of the cases, respectively; and in the dermis a mononuclear inflammatory infiltrate formed mainly by lymphocytes followed by macrophages with very few plasma cells and scarce parasitism was observed [12].

The histopathological aspects of the skin lesion reported in the NUCL [12] differ from those described for cutaneous lesions caused by dermotropic species of the parasite in the New World, such as *L.* (*L.*) *amazonensis*, *L.* (*V*.) *braziliensis* [15], *L.* (*V*.) *guyanensis* [25], and *L.* (*V*.) *panamensis* [26] which are characterized by evident changes in the epidermis that show the presence of ulcer, acanthosis, exocytosis, parakeratosis, and hyperplasia; and mononuclear inflammatory infiltrate varying the intensity and cellular type, granulomatous reaction and necrosis according to the time of infection and the parasite specie. These findings reinforce the role of the parasite in determining the clinical and immunohistopathological features of the infection [16]. In this sense a study using different species of viscerotropic and dermothropic parasites reported that keratinocytes are important nontraditional immune cells that shape the local immune response to *Leishmania* species after their introduction into the skin and in concert with other local factors may contribute to development of different clinical forms of leishmaniasis [27]. Another study reported that Th1 cytokines and keratinocyte growth factor play a critical role in pseudoepitheliomatous hyperplasia indicate that the regulation of leukocyte activation and its recruitment plays a fundamental role in the production and maintenance of this epidermis lesion, through the production of TNF-α and IFN-γ [28]. 

In order to deep knowledge regarding to the *in situ* cellular immune response in the NUCL, the present study evaluate some parameters of the cellular immune response with special reference to lymphocytes subsets by immunohistochemistry. It is important to note that little is known about this clinical form caused by *L.* (*L*.) *infantum chagasi* in Central America [4]; in this way, this study contributes substantially to knowledge about the pathogenesis of this rare clinical form of human infection cause by viscerotropic strain of parasite. The immunohistochemical analysis of the lesions evidenced the presence of a mononuclear infiltrate in the dermis, mainly lymphocytes, characterized by the presence of CD8^+^ T cells, CD4^+^ T cells, NK lymphocytes (CD56^+^) and B lymphocytes (CD20^+^). Macrophages (CD68^+^) were also present in considerable numbers but in lower cell density than CD8^+^ T lymphocytes. These results confirm the findings already reported [12] that describe the presence of mononuclear inflammatory infiltrate, mainly formed by lymphocytes and to a lesser extent by macrophages.

We observed a strong and positive correlation between CD4^+^ T and CD8^+^ T cells and a ratio between these cell types of 0.38, showing that individuals affected by NUCL have a significantly higher number of CD8 T lymphocytes in relation to CD4 T lymphocytes. However, Da Cruz et al. [29,30] showed that in individuals affected by cutaneous leishmaniasis caused by dermotropic strain, *L.* (*V*.) *braziliensis*, the CD4:CD8 ratio was 2.5 in active disease and only observed a ratio of 0.8 during and at the end of the treatment, suggesting that CD8^+^ T lymphocytes could be involved in the healing process.

Positive correlation of CD8^+^ T and CD4^+^ T cells with IFN-γ^+^ cells was observed in our study, suggesting that in NUCL, these cellular types could play an important role in the production of IFN-γ^+^, mainly CD8^+^ T lymphocytes since they are the predominant cells in the inflammatory infiltrate and are presented in equivalent numbers of IFN-γ^+^ cells. IFN-γ^+^ is the main cytokine activator of macrophages that, once activated, are able to produce toxic metabolites and control parasitism, thus showing that CD8^+^ T cells could play a protective role in this rare cutaneous form of leishmaniasis. The ability of CD8^+^ T cells to contribute to the protective or pathological mechanisms in cutaneous leishmaniasis is directly associated with its effector functions. Thus, CD8^+^ T cells are protective when they produce IFN-γ, which activates macrophages to lyse parasites but are associated with tissue injury when they exert cytolytic or cytotoxic activity [31,32]. Studies have shown that in cutaneous and mucocutaneous leishmaniasis caused by *L.* (*V*.) *braziliensis*, CD8^+^ T cells contribute to the exacerbation of the disease due to their cytolytic function, and the severity of the disease in these patients is directly associated with the increase in the number of CD8^+^ T cells expressing granzyme [31-34]. On the other hand, there is evidence that CD8^+^ T cells are essential for the control of primary and secondary infection, since activated CD8^+^ T cells produce chemokines and are an important source of IFN-γ, which modulates granuloma formation and contributes to the reduction in parasitic load [32,34]. These data are similar to those observed in our study, since the inflammatory process of the skin lesion led to granuloma formation in 60% of the cases with discrete tissue parasitism in 100% of the cases. Since the parasitism of skin lesions is very low, as already reported by our group [12], no correlation between parasite load and intensity of the inflammatory infiltrate, evolution time, and the density of the different markers studied was observed.

In PKDL lesion, that is clinically characterized by the presence of hypopigmented macules, erythematous papules and nodules on the skin, and histologically by inflammatory infiltrate in the dermis with consist of a mixture of lymphocytes, histiocytes and plasma cells; IL-10-producing CD3^+^CD8^+^ lymphocytes are important protagonists in the immunopathogenesis [35,36]. It was shown through up regulated IL10 production by CD8^+^ T cells in PKDL patients and some other studies has also provided evidence for the role of IL-10 producing TGF-β in immunopathogenicity which can regulate the IFN-γ dominant protective Th1 response in patients both in peripheral blood mononuclear cells and in dermal lesions of PKDL patients. Additionally, there are reports on a mixed T cell response in PKDL patients since they presented upregulated production of both IL-10 and TNF-α [35,37].

A very interesting finding in our study is that the number of macrophages (CD68^+^ cells) was equivalent to the number of iNOS^+^ cells, the enzyme responsible for the production of nitric oxide, a molecule with leishmanicidal activity, which suggests that all the macrophages in the lesion are likely activated, however, further studies using double staining are still needed. In addition to this result, we found a positive correlation between CD68^+^ cells and iNOS^+^ cells, as well as between CD68^+^ cells and IFN-γ^+^ cells, which could suggest the joint participation of elements that contribute to cellular activation and control of tissue parasitism [38,39]. These results point to a very effective local cellular immune response, since the lesions observed in all patients are small and scare parasitism, regardless of the intensity of the infiltrate, the presence of granulomas and the time of evolution [12].

Th1-type CD4^+^ T lymphocytes are also able to secrete IFN-γ among other inflammatory cytokines, such as IL-2 and TNF-α, activating the cellular immune response towards healing and protection. On the other hand, Th2-type CD4^+^ T lymphocytes secrete anti-inflammatory cytokines, such as IL-4, IL-5, IL-6, IL-10 and IL-13, targeting the cellular immune response of the host to susceptibility to infection [40,41].

Conversely, our previous report using the same biopsies of this study showed very few numbers of IL-10^+^ cells, which did not present correlation to T CD4^+^ cells [14]; so, the present results suggest a participation, albeit discreet, of Th1-type CD4^+^ T cells producing IFN-γ contributing to infection control. Corroborating this interpretation, our results showed a discrete participation of B lymphocytes (CD20^+^ cells) in the NUCL, cells that proliferate and produce immunoglobulins by stimulation of Th2 type CD4^+^ T lymphocytes [42-44]. In addition, we showed that patients with NUCL produce very little antibody, since only 19% of them were seropositive and always had low titers of specific IgG or IgM antibodies. On the other hand, 56% of these patients showed a strongly positive intradermal Montenegro reaction [23], reinforcing the findings of an effective cellular immune response of the host against *L.* (*L*.) *infantum chagasi*.

Despite the efficient response in the skin of individuals affected by NUCL, the total control of the tissue parasitism and the spontaneous healing of the lesion do not occur. The discrete parasite persistence is likely linked to the participation of regulatory cells through the *in situ* production of TGF-β in the lesion of NUCL [14].

## Conclusion

In summary, this study characterize the main mononuclear inflammatory cells present in dermal cutaneous lesions caused by *L. (L.) infantum chagasi,* which were formed mainly by CD8^+^ T lymphocytes, followed by macrophages mostly iNOS^+^, a response that could be mediated by the main inflammatory cytokine, IFN-γ. The results highlight a pivotal contribution of CD8^+^ T lymphocytes in orchestrating host protection against *L.* (*L*.) *infantum chagasi* infection in the skin and add new knowledge on the immunopathology of NUCL. Nevertheless, further studies are necessary to corroborate the protective involvement of CD8 T lymphocytes in the immunopathogenesis of NUCL.
